# Dietary guideline adherence during preconception and pregnancy: A systematic review

**DOI:** 10.1111/mcn.12916

**Published:** 2019-12-02

**Authors:** Cherie Caut, Matthew Leach, Amie Steel

**Affiliations:** ^1^ Office of Research Endeavour College of Natural Health Queensland Australia; ^2^ Department of Rural Health, Division of Health Sciences University of South Australia Whyalla South Australia Australia; ^3^ Australian Research Centre in Complementary and Integrative Medicine, Faculty of Health University of Technology Sydney Ultimo New South Wales Australia

**Keywords:** diet, dietary intake assessment, dietary recommendations, preconception nutrition, pregnancy and nutrition, systematic review

## Abstract

The aim of this study is to determine the level of adherence to dietary guidelines among men and women during preconception, and pregnant women, and factors associated with adherence. Searches were conducted in CINAHL, AMED, EMBASE, and Maternity and Infant Care from inception to March 2018. Observational studies assessing the primary outcome (adherence to dietary guidelines and/or nutritional recommendations) and/or secondary outcome (factors associated with adherence) were eligible. Study quality was assessed using the National Institutes of Health Quality Assessment Tool for Observational Cohort and Cross‐sectional studies. Men or women (aged ≥18 years) who identified as trying/intending to conceive or were pregnant. Eighteen studies were included. The quality of studies was fair (44%) to good (56%). Most studies indicated preconceptual and pregnant women do not meet recommendations for vegetable, cereal grain, or folate intake. Pregnant women did not meet iron or calcium intake requirements in 91% and 55% of included studies, respectively, and also exceeded fat intake recommendations in 55% of included studies. Higher level education was associated with improved guideline adherence in pregnant women, whereas older age and non‐smoking status were associated with greater guideline adherence in preconceptual and pregnant women. The findings of this review suggest that preconceptual and pregnant women may not be meeting the minimum requirements stipulated in dietary guidelines and/or nutritional recommendations. This could have potential adverse consequences for pregnancy and birth outcomes and the health of the offspring. Major knowledge gaps identified in this review, which warrant further investigation, are the dietary intakes of men during preconception, and the predictors of guideline adherence.

**Key messages**

- Dietary guidelines exist to promote health and well‐being through the prevention of diet‐related disorders and nutritional deficiencies.
- Non‐adherence during the preconception period and pregnancy may have a negative impact on fertility, pregnancy and birth outcomes, and the health of the offspring.
- Sociodemographic factors are associated with compliance to dietary guidelines.
- Understanding the factors associated with adherence and non‐adherence can inform future policy and practice aimed at improving overall dietary guideline compliance, during the preconception period and pregnancy.
- Nutrition education and counselling may improve compliance; however, further studies are needed to determine how the intervention translates to maternal and infant health outcomes.

## 1 INTRODUCTION

Dietary guidelines and nutritional recommendations aim to provide guidance on dietary intake/composition and macronutrient/micronutrient intake, respectively. These guidelines serve to maintain health and well‐being and to prevent diet‐related disorders and nutritional deficiencies (World Health Organization [WHO], 2015). The World Health Organization (WHO) and the Food and Agricultural Organization (FAO) of the United Nations developed the estimated average requirement (EAR) and recommended nutrient intake (RNI) as targets for human nutrient intake. EARs meet the average daily nutritional needs of 50% of healthy individuals by age and gender, and RNIs meet the average nutritional needs of nearly all healthy individuals by age and gender and are set as the EAR plus two standard deviations (WHO and Food and Agriculture Organization of the United Nations, 2004). Some countries describe values specific to their populations, such as Recommended Dietary Allowance (RDA; United States, Canada, and United Kingdom) or Recommended Dietary Intake (RDI; Australia; National Health and Medical Research Council [NHMRC], 2013) that are equivalent to the WHO/FAO RNI. Some nutrient intake targets are described as either an acceptable intake (AI) instead of a RNI/RDA/RDI where an EAR has not been established or an upper limit (UL) where excessive intake of the nutrient may cause adverse effects on health. Many countries utilize nutrient EAR, RNI, AI, and UL as a foundation to develop national food‐based dietary guidelines, where foods are grouped and recommendations are made with reference to daily food group servings in an attempt to meet estimated nutrient EAR, RNI, AI, and UL from the diet. Although food group serving suggestions only provide an estimate of EAR, RNI, AI, and UL due to their variant composition, they enable a simplified approach to providing populations with food‐based dietary guidelines that attempt to meet daily nutritional needs (World Health Organization and Food and Agriculture Organization of the United Nations, 2004). Although WHO recommends that all countries adhere to these guidelines (WHO, 2015), non‐adherence remains an ongoing issue across the globe (Development Initiatives, 2017; WHO, 2017) with one in three persons experiencing undernutrition, malnutrition, nutritional deficiencies, or obesity (WHO, 2017).

Multiple factors can impact guideline adherence, including income, food availability and affordability, individual beliefs and preferences, cultural traditions, and educational, social, geographical, and environmental aspects (WHO, 2015). This sociocultural context can exert considerable influence on a diet at different stages of life; in particular, during conception and pregnancy (Withers, Kharazmi, & Lim, 2018). Poor adherence to dietary guidelines or nutritional recommendations during the preconception period (i.e., the weeks to months before pregnancy occurs) and throughout pregnancy can have a negative impact on fertility, pregnancy and birth outcomes, as well as the future health of the offspring (Craig, Jenkins, Carrell, & Hotaling, 2017; Lane, Robker, & Robertson, 2014; Stephenson et al., 2018). For example, in women, adequate intake of folate during the preconception period may help to prevent neural tube defects in the foetus (De‐Regil, Peña‐Rosas, Fernández‐Gaxiola, & Rayco‐Solon, 2015; WHO, 2017). Parental dietary factors are also implicated in the development of disease in the offspring (Fleming et al., 2018). Preventable non‐communicable diseases in the offspring, including cardiovascular diseases, diabetes, cancer, chronic respiratory diseases, atopic disease, and neurological disorders, have been attributed to parental health (physiology, metabolic factors, and body composition) and nutrition status at the time of conception (Fleming et al., 2018). During pregnancy, a healthy diet may prevent excessive weight gain and the associated risks of gestational diabetes, hypertension, or pre‐eclampsia (Schoenaker, Soedamah‐Muthu, Callaway, & Mishra, 2015; Stephenson et al., 2018). A healthy diet also may assist in the prevention of low birth weight infants, macrosomia, preterm birth, stillbirth, and maternal anaemia (World Health Organisation, 2016).

Although non‐adherence to national dietary guidelines and nutritional recommendations is a global issue (WHO, 2015), it is not clear to what extent non‐adherence applies to individuals in the preconception period and during pregnancy. Understanding the level of concordance with dietary guidelines in these populations, as well as the factors impacting adherence, may help to inform the development of future policies and/or practices specifically targeted at improving dietary practices during the preconception period and throughout pregnancy.

## 2 METHODS

This systematic literature review was conducted and reported in accordance with the preferred reporting items for systematic reviews and meta‐analysis: the PRISMA statement (Moher, Liberati, Tetzlaff, & Altman, 2009).

### 2.1 Objectives

The aim of this systematic review was to determine the level of adherence to national/international dietary guidelines in their entirety, among adult men and women during the preconception period, and in pregnant women, and the factors associated with adherence.

### 2.2 Search strategy

Literature searches were conducted in five electronic databases from their inception to March 22, 2018. Table 1 shows the search terms used and the number of articles retrieved in PubMed [NCBI]. A similar search was carried out in CINAHL Plus with Full Text [EBSCOhost], AMED [EBSCOhost], EMBASE [Ovid], and Maternity and Infant Care [Ovid], with some variations to search terms required to accommodate for differences in keywords or subject headings. The reference lists of included papers were also manually searched to detect relevant studies not identified in the electronic search. There were no restrictions on date of publication, study setting, or language.

**Table 1:** PubMed search strategy

No.	Search	Results
1	Preconception care [MeSH] OR preconceptual OR periconceptual OR “trying to conceive”	2,659
2	Pregnant women [MeSH] OR antenatal OR “prenatal care” OR maternity	146,042
3	Subfertility [MeSH] OR fecund^a^	98,227
4	Diet habit [MeSH] OR “diet quality” OR “dietary intake” OR “dietary assessment” OR diet records [MeSH] OR “dietary patterns” OR “dietary habits” OR food diaries [MeSH] OR “food habits” OR “food survey” OR “food intake” OR “food frequency” OR “dietary practices” OR nutrition OR “nutritional intake” OR “nutrition survey”	578,150
5	Dietary guidelines [MeSH] OR “food policy” OR “nutrition guidelines” OR “nutrition policy” OR “dietary recommendations” OR “dietary reference intake” OR “eating guidelines” OR “daily recommended allowance” OR “daily recommended intake” OR “reference daily intake.”	14,015
6	1 OR 2 OR 3	243,954
7	4 AND 5 AND 6	412

a Denotes that this word was truncated during the search string being entered into the electronic databases.

### 2.3 Eligibility

#### 2.3.1 Types of studies

As the review focussed on usual dietary intake and habits, it only considered for inclusion observational studies. Excluded from the review were qualitative and experimental studies.

#### 2.3.2 Types of participants

Studies involving (a) adult men and/or women, aged 18 years or older, that identified as trying to conceive or intending to conceive (i.e., during the preconception period), or (b) pregnant women aged 18 years or older, were eligible for inclusion. Studies involving participants with known preexisting conditions such as polycystic ovary syndrome, obesity, and gestational diabetes were excluded as these populations were likely to be receiving dietary interventions to manage their condition. Obese populations were also likely to have experienced difficulties adhering with dietary guidelines. Also excluded were studies specifically examining participants from known only low or only medium‐high socioeconomic cohorts. These studies were excluded to mitigate contributions to the data with potential to result in skewed effects. Studies examining women's dietary intake throughout the post‐partum period or during lactation were excluded as these groups were not the focus of this review; the exception being if these participants were clearly distinguishable from preconceptual or pregnant populations if reported within the same study.

#### 2.3.3 Types of exposures

Studies observing the usual dietary and/or nutritional intake, and/or dietary habits of participants, the components of which were derived from foodstuffs and beverages, were eligible for inclusion. Studies assessing nutrient intakes arrived from supplement use were excluded, as were studies examining isolated or limited numbers of nutrients (i.e., omega‐3 fatty acids, iodine, calcium, or iron) or limited components of the diet (i.e., total energy or fibre), as these studies cannot be compared with dietary guidelines to determine overall adherence. Also excluded were studies involving participants receiving parenteral nutrition, feeds via percutaneous gastrostomy tubes, and those with special dietary needs (i.e., due to allergy).

#### 2.3.4 Types of outcome measures

The primary outcome was the level of adherence to national/international dietary guidelines and/or recommendations in full; this could be reported using any measure of adherence. The secondary outcome was the factors associated with the level of guideline adherence; this was not limited to any type of factor or association.

#### 2.3.5 Study selection

**Figure 1:**
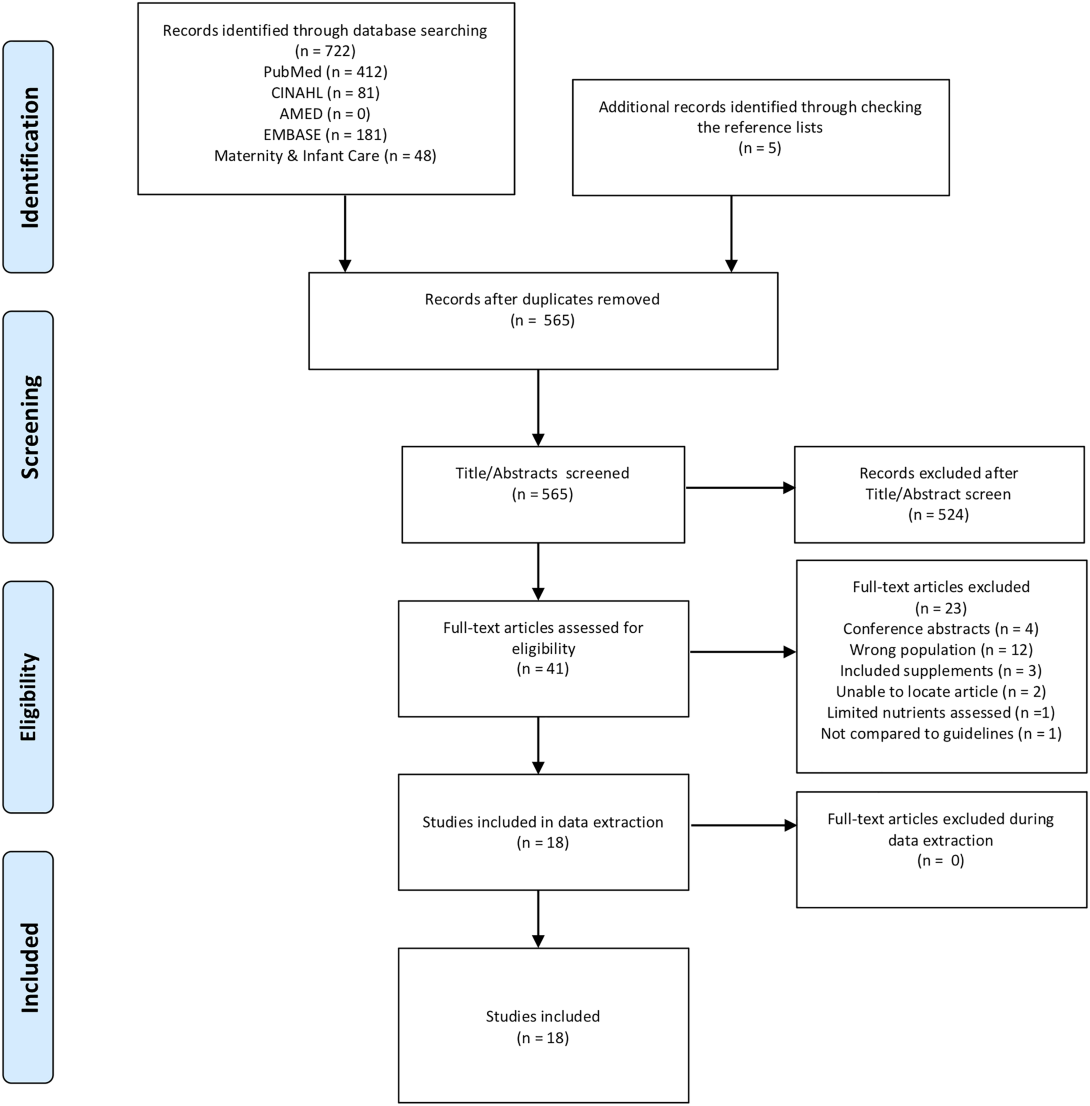
PRISMA flow diagram

The title and abstract of all articles were screened by the lead reviewer (CC) against the predefined inclusion and exclusion criteria (Figure 1). Potentially eligible articles were retrieved as full‐text and screened by CC. Two second reviewers (ML or AS), with methodological and content expertise, used the liberal accelerated approach (Khangura, Konnyu, Cushman, Grimshaw, & Moher, 2012) to randomly review 10% of included studies and 10% of excluded studies. Any disagreements were discussed until consensus was reached. If unresolved, another reviewer (ML or AS) was invited to adjudicate (Figure 1).

#### 2.3.6 Data extraction

Data from each eligible study were extracted by CC using a customized data extraction form. Data extracted from a random selection (10%) of included studies were cross‐checked by a second reviewer (ML or AS) using the liberal accelerated approach, as described previously. The data extracted included author, setting, location, sample characteristics, eligibility, duration of study, study design, exposure, instrument used, main outcome measures, and results (Table 2).

**Table 2:** Characteristics of included studies

Author (year)	Setting	Location	Sample characteristics	Eligibility	Duration of study	Study design	Exposure	Instrument used	Main outcome measure	Result	Risk of bias (critical appraisal)
Melek et al. (2015)	National cohort—online; South Australian cohort (Women's Children's Hospital, Adelaide)	Australia South Australia (Adelaide)	National cohort *n* = 455; South Australian cohort *n* = 402; Total *n* = 857 Sex: Female Mean age: 31.1 years 57% completion rate (857 pregnant women completed the survey out of a potential of 1,493)	Pregnant women living in Australia	June 2013–November 2013 (6 months)	Web‐based cross sectional survey design	Dietary intake of an average week during pregnancy	Self‐administered online 6‐item food frequency questionnaire (FFQ), plus additional questions to collect data pertaining to socioeconomic, attitudes, and knowledge.	(a) Adherence to national dietary recommendations (Australian Dietary Guidelines 2013 [ADG‐2013] for pregnant women) in pregnancy. The number of servings of each of the five food groups consumed per week on average compared with serving recommendations of the five food groups for pregnant women. (b) Factors associated with adherence to the ADG‐2013	56% met fruit servings recommendations; 29% met dairy servings recommendations; <10% met minimum servings recommendations for grains, vegetables, legumes and beans, lean meat and poultry, fish, eggs, nuts and seeds Living outside of the metropolitan area, not smoking, having a healthy BMI, and an annual income >$20,000 AUD are predictors of adherence national pregnancy to dietary guidelines.	Quality rating score: Good
Sajjad et al. (2012)	Antenatal care units of Lady Reading Hospital and Alkhidmat Hospitals Peshawar, Pakistan	Pakistan, Peshawar	*n* = 198 pregnant women. Aged 18–35 years. 66 pregnant women from each trimester of pregnancy. Age distribution 19–30 years (76%) ≥30 years (18%)	Pregnant women age 18–35 years	Unknown	Cross‐sectional survey	24‐hr dietary recall	Interview using a structured questionnaire with 24‐hr dietary intake recall and biographic and socioeconomic questions.	(a) Dietary intake of pregnant women. Assessed using food composition tables and comparison of nutrient intake to World Health Organization (WHO) Recommended Dietary Allowance (RDA) appropriate by age. (b) Association between socioeconomic status and meeting the WHO RDA.	Mean intakes of iron, calcium, zinc, folate, and B12 intake from food did not meet WHO RDA in any age group or trimester of pregnancy. Mean protein intake did not meet WHO RDA in any age group of the first trimester of pregnancy. Mean energy intakes exceeded WHO RDA in all age groups and trimesters except the first trimester of 19–30 years of age group. Nutrient intakes were higher overall in high socioeconomic status pregnant women compared to low socioeconomic status pregnant women.	Quality rating score: Fair
Bookari et al. (2017)	Five antenatal clinics in NSW Hospitals; pregnancy/baby expositions; pharmacies; two NSW retail baby stores	Australia, New South Wales (NSW)	*n* = 388 pregnant women aged 20–40 years. 400 out of 472 completed the survey (84% completion rate). *n* = 12 were excluded due to age <20 years Age distribution: 20–29 years (50%) 30–39 years (46%) ≥40 years (4%)	Pregnant women >20 years	October 2012–July 2013	Cross‐sectional survey completed online	Dietary intake during pregnancy	Self‐administered online validated FFQ. Questions asked the number of servings of five food groups plus extras.	(a) Dietary intake assessed against Australian Guidelines to Healthy Eating (AGHE); the five food groups. (b) Factors that influence adherence to the guidelines. (c) Attitudes towards pregnancy specific nutrition recommendations.	Nil participants met all of the AGHE recommendations of the five food groups during pregnancy. The majority of pregnant women did not meet servings recommendations for vegetables, fruit, breads, and cereals; conversely a large number of pregnant women exceeded servings recommendations of meat and its alternatives and dairy foods. Women knowledgeable about the AGHE five food groups and in their first trimester are more likely to meet recommendations. Women believing a healthy diet was important or extremely important in pregnancy were less likely to adhere to meat servings recommendations.	Quality rating score: Good
Bojar et al. (2006)	Antenatal clinics in Lublin, Poland	Poland, Lublin	*n* = 150 pregnant women 124 out of 150 responded to the survey; 82.7% completion rate. *n* = 119 fully completed. Age range not specified.	Pregnant women	January 2006 (1 month)	Cross‐sectional survey	Consumption of food before pregnancy and during pregnancy	Self‐administered personally designed questionnaire to determine the average consumption of particular groups pf products before and during pregnancy plus biographic and socioeconomic questions	(a) Change in diet from prepregnancy to pregnancy. Compared with WHO nutrition pyramid (meat, dairy products, fruit, vegetables, and crop products). (b) Socioeconomic and environmental factors affecting diet quality.	Compared with the WHO nutrition pyramid, excess diary and inadequate crop and vegetable groups were consumed prior to pregnancy. During pregnancy fruit intake was reduced and inadequate, dairy was excess when compared with WHO nutrition pyramid recommendations. Education, residence (non‐metropolitan), and age were associated with the change in the amount of fruit consumed. Prepregnancy diets were less aligned with the WHO nutrition pyramid than pregnancy diets.	Quality rating score: Fair
Gao et al. (2013)	Antenatal clinics in two hospitals and five rural clinics	China, Deyang City, Sichuan Province	*n* = 203 pregnant women in the 3rd trimester of pregnancy (62.7% urban, 37.3% rural) Aged 19–42 years Age distribution not reported. 201 of 203 completed the survey (99% completion rate)	Pregnant women in the 3rd trimester	July 2010–October 2010 (4 months)	Cross‐sectional study	Previous day food intake according to usual routine	Semistructured interview and 24‐hr dietary recall. Self‐reported height and prepregnancy weight.	(a) Dietary intake and risk factors for nutritional inadequacy. Compared with Chinese dietary reference intakes (DRIs) with recommended nutrient intake (RNI) or adequate intake (AI) for pregnant women.	RNIs were exceeded for fat intake in both rural and urban pregnant women, energy RNI was met in urban not rural pregnant women. No other RNIs met fulfilment for protein, carbohydrate, vitamins A, C, B1, B2, calcium, magnesium, iron, or zinc. Overall urban pregnant women had higher levels of RNIs than rural pregnant women.	Quality rating score: Fair
Zhang et al. (2017)	Two hospitals in Beijing, China; Peking University Third Hospital and Haidian Maternal & Child Health Hospital	China, Beijing	Pregnant women Aged 25–34 years *n* = 130 preterm group *n* = 381 term group Age distribution: 21–24 years (4.6%) 25–29 years (40.8%) 30–34 years (39.2%) 35–46 years (15.4%)	Preterm group: giving birth <37 weeks of gestation Term group: giving birth >37 weeks <42 weeks of gestation No congenital abnormalities or neurological impairments	December 2012–December 2013 12 months	Case–control study	Dietary intake 1 month before delivery	Interview item FFQ representing most common foods in the Chinese diet.	(a) Dietary intake in Chinese pregnant women. Compared with Chinese dietary reference intakes (DRIs) with focus on meeting recommended nutrient intakes (RNIs) or adequate intakes (AIs) of total energy, five macronutrients and 17 micronutrients. (b) Association between dietary nutrients and preterm birth.	Mean energy intake was below DRIs in preterm birth group. Mean vitamin A, calcium, and iron were well below DRIs in both groups and mean intakes of thiamine, riboflavin, and magnesium were below DRIs in both groups. Mean sodium intake was above DRIs in both groups.	Quality rating score: Good
Pick et al. (2005)	Universities, Medical clinics, physicians' offices and community places in Alberta, Canada	Canada, Alberta	*n* = 49 non‐pregnant control women *n* = 52 pregnant women 20–38 weeks of gestation Aged 21–41 years Mean age 29.8 ± 0.56 years 101 completed the study out of 112 respondents (90% completion rate). The main reason for non‐participation was being too busy.	Non‐pregnant women of reproductive age and pregnant women willing to keep diet record. Women were excluded if they had impaired glucose tolerance, diabetes mellitus, limited physical activity, or currently participating in another study.	Unknown	Case–control study	Dietary intake over four consecutive days including one weekend day.	Self‐administered 4‐day dietary recalls in a diet diary format.	(a) Dietary intake of non‐pregnant reproductive age women and pregnant women. Compared against the Healthy Eating Index (HEI) developed by the U.S. Department of Agriculture (USDA) and the food guide pyramid servings for pregnant women. The HEI score is a total score of 100 comprised 10 dietary components each with a total score of 10.	40% of pregnant women did not meet the minimum recommended number of servings of the food guide pyramid. The mean HEI score was 75 out of 100 for pregnant women. Iron and folate recommended daily intakes from the diet were not met by pregnant women.	Quality rating score: Good
Panwar et al. (1998)	Residences of the study participants	India, Haryana State, Northern India	*n* = 90 rural pregnant women *n* = 45 farming *n* = 45 nonfarming Ages of the participants is not specified	Pregnant women from either farming or nonfarming communities of Haryana State.	Unknown	Cross‐sectional study	Three consecutive days of dietary intake	Interview with a 15‐item questionnaire including general family history and details, dietary habits, cooking practices, and foods consumed. Foods were weighed.	(a) Dietary intake compared against recommended dietary allowance (RDA) of the Indian Council of Medical Research (ICMR). (b) Relationship between income and education and nutrient intakes.	Mean dietary intakes of energy calcium and iron were lower than RDAs for all women. Nonfarming pregnant women had lower mean protein intakes. The mean intake of fat was two times the RDA for all women. Folate and vitamin C intakes were below RDA. A higher level of education was associated with a higher level of nutrient intakes except for niacin.	Quality rating score: Fair

Olmedo–Requena et al. (2018)	Catchment area for local Hospital Virgen de las Nieves University Hospital (UHVN)	Spain	*n* = 1,175 pregnant women 1,175 out of 1,222 completed the study (96% completion rate) Mean age 29.8 years	18 years or older, pregnant, Spanish nationality, singleton pregnancy, 20–22 weeks of gestation. Absence of pathology or physical activity that may affect usual dietary intake.	June 2004–March 2007 (34 months)	Cross‐sectional study	Dietary intake before and during pregnancy	Interview with a 118‐item FFQ. Dietary recall and socioeconomic and lifestyle questions.	(a) Adherence to nutritional recommendations in pregnancy. Compared to the Spanish Society of Community Nutrition recommendations of food groups: cereals, vegetables, fruits, dairy products, and protein foods. (b) Factors associated with adherence to guidelines in prepregnancy and the first half of pregnancy.	Prior to pregnancy 21% did not meet vegetable and cereal recommended intakes, 50% did not meet fruit recommended intakes. During pregnancy adherence to all food types decreased. Protein was consumed in excess before and during pregnancy. Low social class, smokers, low physical activity, and younger age were factors associated with decreased adherence.	Quality rating score: Good
Okubo et al. (2010)	Neyagawa City	Japan, Neyagawa City	*n* = 997 pregnant women aged 18–41 years Age distribution: ≤30 years (46.9%) ≥30 years (53.1%) 5–39 weeks of gestation	Pregnant >18 years Living in Neyagawa City	November 2001–November 2003 (2 years)	Cross‐sectional study	Dietary habits of previous month of pregnancy	Self‐administered diet history questionnaire.	(a) Nutritional adequacy of the dietary patterns of Japanese pregnant women. Compared with dietary reference intakes (DRI) for Japanese in pregnancy	Pregnant women consuming a dietary pattern of “rice, fish, and vegetables” have higher levels of nutritional adequacy compared with DRIs than those consuming a dietary pattern of “meat and eggs” or “wheat products.” Although regardless of dietary pattern, more than half of pregnant women had inadequate intakes of fibre, vitamins B1, B6, folate, magnesium, iron, zinc, total fat, saturated fat, and sodium. The more favourable “rice, fish, and vegetable” dietary pattern was associated with higher education and not smoking.	Quality rating score: Good
Mishra et al. (2014)	Two nationally representative age cohorts of Australian women from the Australian Longitudinal Study on Women's Health	Australia	*n* = 1,999 Pregnant women aged 31–36 years. Mean age 34 years.	Pregnant, female, living in Australia. Age 31–36.	Unknown	Cross‐sectional survey.	Previous 12 months of dietary intake in servings per day.	Self‐administered Diet Questionnaire for Epidemiological Studies (DQES).	(a) Dietary intake of pregnant women cohort. Compared the diets of pregnant women cohort against the 2013 Australian Dietary Guidelines.	Less than 2% of pregnant women attained the recommendation of five daily servings of vegetables. 50% of pregnant women met fruit servings, 22% of pregnant women met diary servings, 10% of pregnant women met meat and alternatives servings, and only 2% of pregnant women met servings for cereals.	Quality rating score: Fair
Jood et al. (2002)	Residences of Haryana State, India	India, Haryana State	*n* = 90 pregnant women Aged 18–45 years Age distribution: ≤25 years (60%) 25–35 years (39%) 35–45 years (1%)	Pregnant living in Haryana State	Summer season (3 months)	Cross‐sectional study	Mean daily dietary intake of food groups and mean daily nutritional intake over three consecutive days in the summer season.	Interview 24‐hr dietary recall	(a) Dietary intake of rural pregnant women from Haryana State Northern India. Compared with Indian recommended dietary intakes (RDI).	Mean intake of milk, milk products, and fats and oils were higher than RDIs. Mean intakes of green leafy vegetables and fruits were lower than RDIs. Mean intake of nutrients of protein, beta‐carotene, iron, thiamine, riboflavin, niacin, and ascorbic acid were below RDI.	Quality rating score: Fair

Hure et al. (2008)	A population‐based cohort participating in the Australian Longitudinal Study on Women's Health	Australia	Australian women aged 25–30 years Pregnant mean age 27.4 ± 1.4 years Trying mean age 27.5 ± 1.4 years Pregnant *n* = 606 Trying to conceive *n* = 454	25–30 years of age, Australian, female, able to state pregnancy status by pregnant or trying to conceive	March 2003 (1 month)	Cross‐sectional study	Usual dietary intake of the past 12 months	Self‐administered. The Dietary Questionnaire for Epidemiological Studies (DQES) version 2. 200‐item food frequency questionnaire (FFQ).	(a). Diet quality by pregnancy status. Expressed as the Australian Recommended Food Score (ARFS) based on dietary guidelines for Australian adults and Australian Guide for Health Eating and the National Health and Medical Research Council of Australia Nutrient Reference Values (NRV), either as an estimated average requirement (EAR) or adequate intake (AI).	Out of a possible total of 72 points for the ARFS as a determinant of diet quality; the mean score for pregnant women and women trying to conceive were 30.2 and 29.4, respectively. This indicates that both pregnant and trying to conceive women are not meeting nationally recommended targets for a healthy diet. As the ARFS increased the nutrient intakes of carbohydrate, fibre, sugars, protein, polyunsaturated fat, calcium, iron, zinc, beta‐carotene, folate, thiamine, riboflavin, niacin, and vitamins E, C, and K increased. Nutrients most consistently below NRVs were fibre, folate, and vitamin E. In pregnant women iron is well below EAR and in trying to conceive women vitamin K was below AI. Sodium however was above recommended upper levels in both groups.	Quality rating score: Good
Dahiya (2002)	Three maternity hospitals from Hisar City: Sewak Sabha Charitable Trust Hospital, HAU Hospital, Sharma Ravin Hospital, and Parashar Maternity and Nursing Home	India, Hisar, Haryana	*n* = 120 pregnant women aged 20–30 years Age distribution: ≤20 years (20%) 20–30 years (76.6%) ≥30 years (3.4%)	Pregnant living in Hisar City in the 2nd and 3rd trimester of pregnancy, not consuming supplements and of cognitive ability to complete dietary recall.	Unknown	Cross‐sectional study	Dietary recall of the past 24 hr	Questionnaire and interview 24‐hr dietary recall.	(a). Nutrient and dietary intakes of pregnant women. Compared with the Indian Council of Medical Research Food Composition tables and recommended dietary allowance (RDA).	Mean servings of cereals, pulses, green leafy vegetables were below RDA. Mean servings of milk, milk products, fruits, roots and tubers, and fats and oils were above RDA. Mean intake of total calories, folic acid, iron, niacin, and riboflavin were below RDA.	Quality rating score: Fair
Pinto et al. (2008)	Antenatal clinics at two public hospitals in Porto, Portugal	Portugal	*n* = 249 pregnant women. Mean age 29 years. Pregnant women recruited at less than 13 weeks of gestation.	Pregnant, 13 weeks of gestation and attending the antenatal clinic And within a few days of delivery	May 2005–August 2006 (16 months)	Cross‐sectional study	Usual dietary intake in frequency of consumption as well as portion size in the year prior to pregnancy and for the duration of pregnancy	Interview delivered semiquantitative FFQ, comprising of 86 food or food group items. Nutrients of foods were determined from Portuguese food composition tables. The FFQ was completed at the first antenatal appointment and a few days after delivery.	(a) Maternal diet and nutritional adequacy prior to conception and during pregnancy. Compared with U.S. daily reference intakes (DRIs) and estimated average requirements (EARs) or adequate intakes (AI).	Both prior to pregnancy and during pregnancy inadequate intake of vitamin E, folate, and magnesium were found. In pregnant women inadequate intake of iron was also found. No clear association was found between social and behavioural characteristics of the participants and intake inadequacy.	Quality rating score: Good
de Weerd et al. (2003)	Local advertisements, health clinics and day care centres in Nijmegen	Netherlands, Nijmegen	*n* = 46 women planning a pregnancy. Age 19–45 years (mean age 31.5 years) *n* = 23 control women *n* = 101 volunteered; 72% completed the questionnaires; four women were excluded: one was pregnant; one was postmenopausal; and two could not be reached to verify answers.	Women of reproductive age 19–45 year not planning a pregnancy or women planning a pregnancy age 19–45 years.	January 2001–June 2001 (6 months)	Case–control study	Foods consumed in the past month.	Self‐administered and verified by phone interview. A validated semiquantitative FFQ. Biographic, socioeconomic data, and health practices information or personal views were obtained through additional questions.	(a) Nutritional intake and lifestyle factors in women planning pregnancy. Compared with Dutch recommended dietary allowance (RDA).	Intakes of iron in 74% of women planning pregnancy, selenium in 59% of women planning pregnancy, vitamin A in 48% of women planning pregnancy, and copper in 91% of women planning pregnancy were below RDA. Mean intakes of energy, saturated fat, protein, and carbohydrate exceeded RDA in women planning a pregnancy. 96% of women planning a pregnancy drunk alcohol, and 20% of them reported smoking.	Quality rating score: Good
Liu et al. (2015)	Service centres of maternal and child care in eight cities across China	China	*n* = 479 pregnant women mean age 27 ± 3.6 years Stage of pregnancy: *n* = 158 in first trimester *n* = 160 in second trimester *n* = 161 in third trimester	Healthy pregnant women aged 20–40 years, living in one of eight cities in urban China, no history of chronic disease, consumption of ≤2 alcoholic drinks per week, and no history of smoking. Pregnant women were excluded if they had gestational diabetes, hypertension, infectious disease, mental disease, recall deficit, or were taking medical drugs.	2011‐2012 (12 months)	Cross‐sectional study	Dietary intake of previous day	Interview and 24‐hr dietary recall	(a) Nutrient intake of Chinese pregnant women. Compared with Chinese dietary reference intakes (DRIs) 2013, Chinese recommended nutrient intake (RNI), and estimated average requirements (EARs). (b) Factors associated with the nutrient intakes of urban Chinese pregnant women.	Across all trimesters of pregnancy, the mean/median intake of vitamins A, B6, calcium, magnesium, and selenium were below RNI and EAR. Thiamine was below EAR in all trimesters. Iron intake in the third trimester was below RNI and EAR. The majority of pregnant women had excessive energy intake from fat. Mean sodium intake was above adequate intake levels.	Quality rating score: Fair
Yang et al. (2016)	Shaanxi Province	China	*n* = 7462 pregnant women or women that had delivered less than 12 months prior with aged 25–29 years. Age distribution: ≤25 years (41%) 25–29 years (37.6%) ≥30 years (20.6%) *n* = 7,750 were recruited. *n* = 288 were excluded due to reporting excessive dietary energy intakes. The mean time frame of reporting on dietary intake for women that had already delivered was 3 months.	Pregnant women or women that had delivered less than 12 months prior. Exclusion of women with limited cognitive ability.	August 2013–November 2013 (16 months)	Cross‐sectional study	Dietary intake of entire pregnancy	Interview semiquantitative FFQ.	(a) Nutrient intakes of Chinese women during pregnancy. Compared with Chinese dietary intakes 2013, recommended nutrient intake (RNI), and adequate intake (AI) for pregnant women. (b) Dietary patterns of Chinese women during pregnancy. (b) Association with socioeconomic characteristics of pregnant women and nutrient intakes.	Mean intakes of vitamin A, folate, calcium, and zinc were below RNI. Mean intakes of fat, niacin, and vitamin E were above RNI. Three dietary patterns: balanced pattern, vegetarian pattern, and snacks pattern were identified. The balanced dietary pattern was associated with increased nutrient intakes compared with vegetarian or snacks pattern. The balanced dietary pattern was associated with education, income, residential location, and age.	Quality rating score: Good

#### 2.3.7 Critical appraisal

The lead reviewer (CC) and a second reviewer (ML or AS) critically appraised each included study, independently, using the National Institutes of Health Quality Assessment Tool for Observational Cohort and Cross‐sectional studies (National Heart, Lung and Blood Institute (NHBLI), 2018). The appraisal tool comprised 14 items, with each item rated as either yes (criterion met), no (criterion not met), not applicable, cannot determine, or not reported. An overall quality rating (i.e., good, fair, and poor) was then subjectively determined by the reviewer (CC; Table 3).

**Table 3:** Critical appraisal of included studies

	Malek et al. 2015	Sajjad et al. (2012)	Bookari et al. (2017)	Bojar et al. (2006)	Gao et al. (2013)	Zhang et al. (2017)	Pick et al. (2005)	Panwar et al. (1998)	Olmedo–Requena et al. (2018)	Okubo et al. (2010)	Mishra et al. (2014)	Jood et al. (2002)	Hure et al. (2008)	Dahiya (2002)	Pinto et al. (2008)	de Weerd et al. (2003)	Liu et al. (2015)	Yang et al. (2016)
Was the research question or objective in this paper clearly stated?	Yes	Yes	Yes	Yes	Yes	Yes	Yes	Yes	Yes	Yes	Yes	Yes	Yes	Yes	Yes	Yes	Yes	Yes
Was the study population clearly specified and defined?	Yes	Yes	Yes	Yes	Yes	Yes	Yes	CD	Yes	Yes	Yes	Yes	Yes	Yes	Yes	Yes	Yes	Yes
Was the participation rate of eligible persons at least 50%?	Yes	CD	Yes	Yes	Yes	Yes	Yes	NR	Yes	No	NR	NR	No	NR	Yes	Yes	NR	Yes
Were all the subjects selected or recruited from the same or similar populations (including the same time period)? Were inclusion and exclusion criteria for being in the study prespecified and applied uniformly to all participants?	Yes	CD	Yes	CD	NR	Yes	Yes	Yes	Yes	Yes	No	Yes	Yes	CD	Yes	Yes	Yes	Yes
Was the sample size justification, power, description, or variance and effect estimates provided?	Yes	NR	Yes	Yes	Yes	NR	NR	NR	NR	Yes	NR	NR	NR	NR	NR	NR	Yes	NR
For the analyses in this paper, were the exposure(s) of interest measured prior to the outcome(s) being measured?	Yes	Yes	Yes	Yes	Yes	Yes	Yes	Yes	Yes	Yes	Yes	Yes	Yes	Yes	Yes	Yes	Yes	Yes
Was the timeframe sufficient so that one could reasonably expect to see an association between exposure and outcome if it existed?	Yes	No	Yes	Yes	No	Yes	Yes	Yes	Yes	Yes	Yes	Yes	Yes	No	Yes	Yes	No	Yes
For exposures that can vary in amount or level, did the study examine different levels of the exposure as related to the outcome (e.g., categories of exposure or exposure measured as continuous variable)?	Yes	Yes	Yes	Yes	Yes	Yes	Yes	Yes	Yes	Yes	Yes	Yes	Yes	Yes	Yes	Yes	Yes	Yes
Were the exposure measures (independent variables) clearly defined, valid, reliable, and implemented consistently across all study participants?	Yes	Yes	Yes	Yes	Yes	Yes	Yes	Yes	Yes	Yes	Yes	Yes	Yes	Yes	Yes	Yes	No	Yes
Was the exposure (S) assessed more than once over time?	No	No	No	Yes	No	No	Yes	Yes	Yes	No	No	Yes	No	No	Yes	No	No	No
Were the outcome measures (dependent variables) clearly defined, valid, reliable, and implemented consistently across all study participants?	Yes	Yes	Yes	Yes	Yes	Yes	Yes	Yes	Yes	Yes	Yes	Yes	Yes	Yes	Yes	Yes	Yes	Yes
Were the outcome assessors blinded to the exposure status of participants?	NA	NA	NA	NA	NA	NA	NA	NA	NA	NA	NA	NA	NA	NA	NA	NA	NA	NA
Was loss to follow‐up after baseline 20% or less?	NR	NR	NR	NR	NA	Yes	Yes	NA	Yes	NR	NR	NR	NR	NR	Yes	No	NR	Yes
Were key potential confounding variables measured and adjusted statistically for their impact on the relationship between exposure (S) and outcome (S)?	Yes	Yes	Yes	Yes	No	Yes	Yes	CD	Yes	Yes	CD	CD	Yes	NR	Yes	Yes	Yes	Yes
Quality rating (Good, Fair, Poor)	Good	Fair	Good	Fair	Fair	Good	Good	Fair	Good	Good	Fair	Fair	Good	Fair	Good	Good	Fair	Good
Rater #1 initials:	CC	CC	CC	CC	CC	CC	CC	CC	CC	CC	CC	CC	CC	CC	CC	CC	CC	CC
Rater #2 initials:					AS				AS			ML					ML	
Additional comments (If poor, please state why):																		
Was the research question or objective in this paper clearly stated?	Yes	Yes	Yes	Yes	Yes	Yes	Yes	Yes	Yes	Yes	Yes	Yes	Yes	Yes	Yes	Yes	Yes	Yes
Was the study population clearly specified and defined?	Yes	Yes	Yes	Yes	Yes	Yes	Yes	CD	Yes	Yes	Yes	Yes	Yes	Yes	Yes	Yes	Yes	Yes

Abbreviations: AS, Dr Amie Steel; CC, Cherie Caut; CD, cannot determine; ML, Dr Matthew Leach; NA, not applicable; NR, not reported.

#### 2.3.8 Data synthesis

Given the descriptive nature of this review, the analysis of included studies was undertaken in a narrative manner.

## 3 RESULTS

Database searches retrieved 722 articles, and an additional five articles were found by manually searching the reference lists of included studies. After the removal of duplicates, 565 articles remained. Of these, 524 were removed at the title/abstract screening stage, and 23 removed at the full‐text screening stage as they did not meet the eligibility criteria (Figure 1). A total of 18 studies met the inclusion criteria for this review.

### 3.1 Characteristics of included studies

The included studies comprised 15 cross‐sectional and three case–control studies. These studies were conducted in Australia (*n* = 4) (Bookari, Yeatman, & Williamson, 2017; Hure, Young, Smith, & Collins, 2009; Malek, Umberger, Makrides, & Zhou, 2016; Mishra, Schoenaker, Mihrshahi, & Dobson, 2015), China (*n* = 4) (Gao et al., 2013; Liu et al., 2015; Yang et al., 2017; Zhang, Zhou, Perkins, Wang, & Sun, 2017), India (*n* = 3) (Dahiya, 2002; Jood, Bishnoi, & Khetarpaul, 2002; Panwar & Punia, 1998), Pakistan (*n* = 1) (Sajjad & Khan, 2012), Poland (*n* = 1) (Bojar, Wdowiak, Humeniuk, & Blaziak, 2006), Canada (*n* = 1) (Pick, Edwards, Moreau, & Ryan, 2005), Spain (*n* = 1) (Olmedo‐Requena et al., 2017), Japan (*n* = 1) (Okubo et al., 2011), Portugal (*n* = 1) (Pinto, Barros, & Santos Silva, 2009), and the Netherlands (*n* = 1) (de Weerd et al., 2003). Seventeen studies reported the dietary intake of pregnant women, and five reported the dietary intake of women in the preconception period. No study reported the dietary intake of men in the preconception period. The majority of included studies (*n* = 16) compared the dietary intakes of participants to national dietary guidelines. Only two studies compared dietary intakes to international dietary guidelines. Nine studies reported on factors predicting guideline adherence (Table 2). The dietary recall method was utilized in seven studies; four of these studies conducted a 24‐hr recall, with the remaining assessing dietary recall over three or four consecutive days. Eleven studies utilized food frequency questionnaires (FFQ) assessed over periods ranging from 1 week to 12 months.

### 3.2 Quality of included studies

The quality of the included studies was fair (44% of studies) to good (56% of studies; Table 3). Six studies (Bojar et al., 2006; Bookari et al., 2017; Gao et al., 2013; Malek et al., 2016; Okubo et al., 2011) reported on sample size justification, and six studies (Bojar et al., 2006; Jood et al., 2002; Olmedo‐Requena et al., 2017; Panwar & Punia, 1998; Pick et al., 2005; Pinto et al., 2009) assessed dietary intake more than once over time. Two studies (Bojar et al., 2006; Panwar & Punia, 1998) did not specify the age of the participants. The authors of these studies were contacted for further information; however, no response was received from any author.

### 3.3 Outcomes

#### 3.3.1 Dietary intake in pregnancy

Of the eight studies reporting dietary intake (as food groups) among pregnant women, most indicated that women during pregnancy were least adherent with dietary recommendations for the daily intake of vegetables (Bookari et al., 2017; Dahiya, 2002; Jood et al., 2002; Malek et al., 2016; Mishra et al., 2015), cereals and grains (Bookari et al., 2017;Dahiya, 2002 ; Malek et al., 2016 ; Mishra et al., 2015), and most frequently adherent with recommendations for dairy (Bojar et al., 2006; Bookari et al., 2017; Dahiya, 2002; Malek et al., 2016; Pinto et al., 2009) and fruit intake (Dahiya, 2002; Malek et al., 2016; Mishra et al., 2015). Of the eleven studies reporting dietary intake (as nutrients) among pregnant women, the consumption of iron (Dahiya, 2002; Gao et al., 2013; Hure et al., 2009; Jood et al., 2002; Liu et al., 2015; Okubo et al., 2011; Panwar & Punia, 1998; Pick et al., 2005; Pinto et al., 2009; Sajjad & Khan, 2012), folate (Dahiya, 2002; Hure et al., 2009; Okubo et al., 2011; Panwar & Punia, 1998; Pick et al., 2005; Pinto et al., 2009; Sajjad & Khan, 2012; Yang et al., 2017), and calcium (Gao et al., 2013; Liu et al., 2015; Panwar & Punia, 1998; Sajjad & Khan, 2012; Yang et al., 2017; Zhang et al., 2017) were considered by most studies as being inadequate. By contrast, fat intake frequently exceeded daily recommended levels (Dahiya, 2002; Gao et al., 2013; Jood et al., 2002; Liu et al., 2015; Panwar & Punia, 1998; Yang et al., 2017).

#### 3.3.2 Dietary intake in the preconception period

Of the two studies reporting dietary intake as food groups, evidence indicated that most women in the preconception period did not achieve the recommended daily intake of vegetables (Bojar et al., 2006; Okubo et al., 2011) and cereals (Bojar et al., 2006; Okubo et al., 2011), but most frequently met requirements for dairy intake (Bojar et al., 2006). Of the three studies reporting dietary intake as nutrients, the consumption of folate (Hure et al., 2009; Pinto et al., 2009) and vitamin E (Hure et al., 2009; Pinto et al., 2009) was most frequently reported as being inadequate in the diets of preconceptual women. On the other hand, protein intake was more likely to exceed daily recommendations (de Weerd et al., 2003; Olmedo‐Requena et al., 2017).

#### 3.3.3 Factors predicting adherence

Nine studies reported on factors predicting guideline adherence in pregnancy. Most of these studies reported a statistically significant association between higher level of education (Bojar et al., 2006; Okubo et al., 2011; Panwar & Punia, 1998; Yang et al., 2017), older age (Bojar et al., 2006; Okubo et al., 2011; Olmedo‐Requena et al., 2017), or non‐smoking status (Malek et al., 2016; Okubo et al., 2011; Olmedo‐Requena et al., 2017) and adherence to dietary guidelines or nutritional recommendations. The impact of residential location on guideline adherence was contradictory; two studies (Bojar et al., 2006; Malek et al., 2016) reported a statistically significant positive association between level of guideline adherence and living in non‐metropolitan residences, whereas two studies (Gao et al., 2013; Yang et al., 2017) found a statistically significant positive association between level of adherence and living in urban residences. Only one study (Olmedo‐Requena et al., 2017) reported on factors associated with guideline adherence among women during the preconception period. This study found a statistically significant association between guideline adherence and higher social class, non‐smoking status, increased physical activity, and older age.

## 4 DISCUSSION

This review synthesized the evidence from observational studies reporting the level of adherence to national/international dietary guidelines and/or nutritional recommendations, and the factors impacting adherence, among women in the preconception period, and during pregnancy. The eighteen studies included in this review were conducted across 10 countries, thus representing a broad perspective on the issue. The review found that overall adherence to dietary guidelines during the preconception period and pregnancy was low, with a number of demographic factors shown to have an impact on guideline adherence.

Evidence from this review suggests that women in the preconception period, and during pregnancy, may not be meeting the minimum dietary requirements for vegetable and cereal grain intake. According to WHO, vegetables and cereal grains should form a part of a healthy diet for the prevention of diet‐related non‐communicable disease and nutritional deficiencies (WHO, 2015). Vegetables and cereal grains are low in fat and key sources of vitamins, minerals, protein, and dietary fibre (National Health and Medical Research Council, 2013). Diets high in grains and vegetables may help reduce the risk of obesity, type 2 diabetes, cardiovascular disease, and cancer (National Health and Medical Research Council, 2013). For preconceptual and pregnancy health, vegetables are an important source of folate, and cereal grains a valuable source of folate and iron (National Health and Medical Research Council, 2013).

The insufficient intake of certain food groups by preconceptual and pregnant women may also impact nutrient intake, with findings of the review suggesting that dietary folate, dietary iron, and dietary calcium intakes may be inadequate in these populations. This is concerning given the body of evidence supporting an association between adequate preconceptual folate intake and lowered risk of neural tube defects (De‐Regil et al., 2015; WHO, 2017). Further, adequate folate intake during pregnancy may reduce the risk of megaloblastic anaemia and help increase birthweight (Lassi, Salam, Haider, & Bhutta, 2013). Adequate iron intake during pregnancy is important for the prevention of adverse pregnancy and birth outcomes, such as maternal anaemia, low birth weight infants, and preterm births (Peña‐Rosas, De‐Regil, Garcia‐Casal, & Dowswell, 2015; World Health Organisation, 2017). The findings of this review are consistent with that of other reports, which indicate that iron deficiency is a global problem (with two billion people worldwide failing to meet the required dietary intake of iron), and the prevalence of anaemia in women of reproductive age is increasing (Development Initiatives, 2017). Maintaining an adequate intake of dietary calcium during pregnancy is also of importance as it may help reduce the risk of pre‐eclampsia (Hofmeyr & Manyame, 2017).

Our findings also indicate that fat intake among pregnant women may exceed recommended levels stipulated in national/international dietary guidelines. However, it is important to note that the included studies assessing fat consumption only reported total fat intake (Liu et al., 2015; Panwar & Punia, 1998; Yang et al., 2017) or the combination of fats and oils (Dahiya, 2002; Gao et al., 2013; Jood et al., 2002). In other words, these studies did not differentiate between the types of fats consumed in the diet, such as saturated fats, trans fats, and omega‐3/omega‐9 fatty acids. In light of emerging clinical evidence linking omega‐3 fatty acid intake in pregnancy to improvements in infant cognitive development (Braarud et al., 2018), and the prevention of allergic disease (Best, Gold, Kennedy, Martin, & Makrides, 2016), this information would have been beneficial. Notwithstanding, the finding that total dietary fat intake exceeded recommendations (i.e., 30% of total energy) is still clinically significant given that this may potentially contribute to unhealthy maternal weight gain (World Health Organisation, 2015). The impact of this weight gain for both the mother and infant can be considerable, with maternal obesity shown in multiple systematic reviews (Marchi, Berg, Dencker, Olander, & Begley, 2015) to be associated with an increased risk of pre‐eclampsia, gestational diabetes, macrosomia, congenital abnormalities, still birth, low birthweight infants, and maternal mortality (Marchi et al., 2015).

The included studies reported three main predictors of adherence to dietary guidelines or nutritional recommendations. Among pregnant women, a higher level of education was the principal predictor of adherence, and among preconceptual and pregnant women, non‐smoking status, and older age were the main predictors. A potential implication of this finding is that consideration needs to be given to how dietary guidelines and recommendations are communicated to different subgroups of preconceptual and pregnant women. Beyond the development of clear and accessible dietary guidelines and nutritional recommendations, consideration could be given to the role of nutrition education and counselling in clinical settings.

Nutrition education and counselling has been shown in a meta‐analysis of 37 studies to be an effective approach for improving maternal nutrition in pregnancy, particularly in reducing maternal anaemia, increasing infant birth weight, and decreasing preterm births (Girard & Olude, 2012). Findings from a randomized controlled trial also indicate that dietary counselling may be effective in decreasing the proportion of women with excessive weight gain during pregnancy through improvements in food knowledge, diversity of food choices, and attitudes to diet (Abdel‐Aziz, Hegazy, Mohamed, Abu El Kasem, & Hagag, 2018). Strategies aimed at improving nutrition and health behaviours before conception also have been explored. One framework recently described by Barker et al. (2018) acknowledges the importance of identifying the age and life phase of an individual; this may help to identify the individual's motivations and likely receptiveness to different intervention types, thus enabling a clinician to deliver a more targeted approach to preconception management (Barker et al., 2018). Additionally, Barker et al. (2018) suggest that an intervention framework should combine public health policy with strategic engagement from the private sector in order to further improve health outcomes (Barker et al., 2018). Of course, further work is needed to determine whether such an approach actually translates into improvements in maternal and infant outcomes.

An important knowledge gap identified through this review is the absence of any studies examining dietary intake in males during the preconception period (i.e., the male partner of a woman trying to conceive), and despite having an important role to play in preconception, men have been largely overlooked in the literature to date (Kotelchuck & Lu, 2017). This represents a much needed area for future research, for several reasons. First, epigenetic changes to the DNA of spermatozoa may be accelerated through nutrition and lifestyle factors, and these changes may have long‐lasting transgenerational effects (Lane et al., 2014). Second, paternal obesity has implications for fertility, in particular, an association with disturbed endocrinology, erectile dysfunction, and increased sperm DNA fragmentation (El Salam, 2018). Indeed, reduced rates of clinical pregnancies in assisted reproductive cycles utilizing sperm of obese men have been observed (Mushtaq et al., 2018). Evidence also suggests that the offspring of obese fathers may result in metabolic changes within the offspring and the subsequent inheritance of obesity (Craig et al., 2017). A better understanding of the nutritional status of males during the preconception period, as well as the factors impacting nutrition in this population, would help to provide a targeted approach for couples experiencing infertility.

Several limitations exist within this review. One limitation is the heterogeneity of instruments used to assess dietary intake. Some studies used dietary recall methods and others FFQs; there was also great variability in reported time frames, ranging from 24 hr to 12 months. These differences in outcomes and outcome measures, and the disparities in how data were reported, meant the pooling of data for meta‐analysis was not possible. Lastly, the dietary intakes reported in the included studies were assessed for adherence against 13 various national food‐based dietary guidelines (i.e., food groups) and/or national or international nutritional recommendations (i.e., RNI/RDA/EAR/AI/UL), dependent upon the country the study was conducted. Given that the recommendations reported in each of these guidelines may differ to some extent, it is possible that adherence (or non‐adherence) to one guideline may not be necessarily transferable to all dietary guidelines. Although beyond the scope of this review, comparisons between countries/regions that utilize RNI/RDA/EAR/AI/UL for nutritional recommendations and food‐based dietary guidelines would be an interesting area for future research to determine how well the two approaches align. For example, a sensitivity and specificity analysis could explore whether populations meeting food group serving recommendations also meet RNI/RDA/EAR/AI/UL recommendations.

## 5 CONCLUSION

The consumption of a healthy diet during the preconception period and pregnancy can be achieved by adhering to national/international dietary guidelines and/or nutritional recommendations. Concordance with these guidelines should provide some assurance that energy, macronutrient, and micronutrient intake are adequate to support fertility, pregnancy, and positive birth outcomes, as well as the future health of the offspring. The findings from this review indicate that women both in the preconception period and throughout pregnancy may be falling short of targets stipulated in dietary guidelines and nutrient recommendations. In particular, preconceptual and pregnant women may not be consuming enough vegetables, cereal grains, folate, iron, and calcium and may be consuming excess fat. A number of demographic factors were shown to impact guideline adherence, which could have implications for both future policy and practice. However, further research is needed to assess the predictors of guideline adherence for both men and women in the preconception period, as well as the quality of diets of men during preconception.

## CONFLICT OF INTEREST

The authors declare that they have no conflicts of interest.

## FINANCIAL SUPPORT

None.

## CONTRIBUTIONS

CC formulated the research question and undertook the database searches, study selection, data extraction, and critical appraisal. AS and ML contributed to the design of the research question, served as second reviewers at the study selection, data extraction, and critical appraisal phases and contributed to manuscript drafting. All authors approved the final manuscript before submission.
